# Applying artificial intelligence to rare diseases: a literature review highlighting lessons from Fabry disease

**DOI:** 10.1186/s13023-025-03655-x

**Published:** 2025-04-17

**Authors:** Dominique P. Germain, David Gruson, Marie Malcles, Nicolas Garcelon

**Affiliations:** 1 Division of Medical Genetics, University of Versailles–St Quentin en Yvelines (UVSQ), Paris–Saclay University, 2 avenue de la Source de la Bièvre, 78180 Montigny, France; 2 First Faculty of Medicine, Charles University, Prague, Czech Republic; 3 Ethik-IA, PariSanté Campus, 10 Rue Oradour-Sur-Glane, 75015 Paris, France; 4 Takeda France SAS, 75116 Paris, France; 5 Imagine Institute, Data Science Platform, INSERM UMR 1163, Université de Paris, 75015 Paris, France

**Keywords:** Artificial intelligence, Machine learning, Deep learning, Rare diseases, Fabry disease

## Abstract

**Background:**

Use of artificial intelligence (AI) in rare diseases has grown rapidly in recent years. In this review we have outlined the most common machine-learning and deep-learning methods currently being used to classify and analyse large amounts of data, such as standardized images or specific text in electronic health records. To illustrate how these methods have been adapted or developed for use with rare diseases, we have focused on Fabry disease, an X-linked genetic disorder caused by lysosomal α-galactosidase. A deficiency that can result in multiple organ damage.

**Methods:**

We searched PubMed for articles focusing on AI, rare diseases, and Fabry disease published anytime up to 08 January 2025. Further searches, limited to articles published between 01 January 2021 and 31 December 2023, were also performed using double combinations of keywords related to AI and each organ affected in Fabry disease, and AI and rare diseases.

**Results:**

In total, 20 articles on AI and Fabry disease were included. In the rare disease field, AI methods may be applied prospectively to large populations to identify specific patients, or retrospectively to large data sets to diagnose a previously overlooked rare disease. Different AI methods may facilitate Fabry disease diagnosis, help monitor progression in affected organs, and potentially contribute to personalized therapy development. The implementation of AI methods in general healthcare and medical imaging centres may help raise awareness of rare diseases and prompt general practitioners to consider these conditions earlier in the diagnostic pathway, while chatbots and telemedicine may accelerate patient referral to rare disease experts. The use of AI technologies in healthcare may generate specific ethical risks, prompting new AI regulatory frameworks aimed at addressing these issues to be established in Europe and the United States.

**Conclusion:**

AI-based methods will lead to substantial improvements in the diagnosis and management of rare diseases. The need for a human guarantee of AI is a key issue in pursuing innovation while ensuring that human involvement remains at the centre of patient care during this technological revolution.

## Background

Most studies about artificial intelligence (AI) and rare diseases were published in the past couple of years, illustrating the rapid development of a new era for accurate screening, management, and care of patients with rare diseases [1–12].

AI is a technology that enables computers to mimic human intelligence and perform numerous complex tasks. Machine learning (ML) is a subset of AI, and deep learning (DL) [12] is a subset of ML that involves more parameters than traditional methods (see Table 1). These techniques are constantly being updated and readers are encouraged to check online resources for the latest changes [13, 14]. The computational power of today's computers has made it possible to develop ML algorithms for rare diseases using three methods: supervised, unsupervised, and reinforcement learning (see Table 1) [1, 2, 15]. Supervised learning involves “labelled data” prepared by humans to train the model and establish the relationship between input and output data, allowing the model to then make output predictions from new input data. Two types of supervised learning can be distinguished: regression, which predicts numerical values (e.g., laboratory values for biochemical tests), and classification, which predicts categorical values (e.g., the presence or absence of genomic variants) (see Table 1). Unsupervised learning involves models that can recognize similarities, recurrent patterns or differences in “unlabelled data” without prior training, allowing patterns and/or relationships to be identified, and data clustering and association analyses to be performed (e.g., image classification or the identification of patients with similar symptoms). Finally, “reinforcement learning” [16] involves techniques that allow the machine to make better decisions over time by following a trial-and-error method and positive or negative feedback approach to improve the final outcome (e.g., text summarization).

**Table 1:**
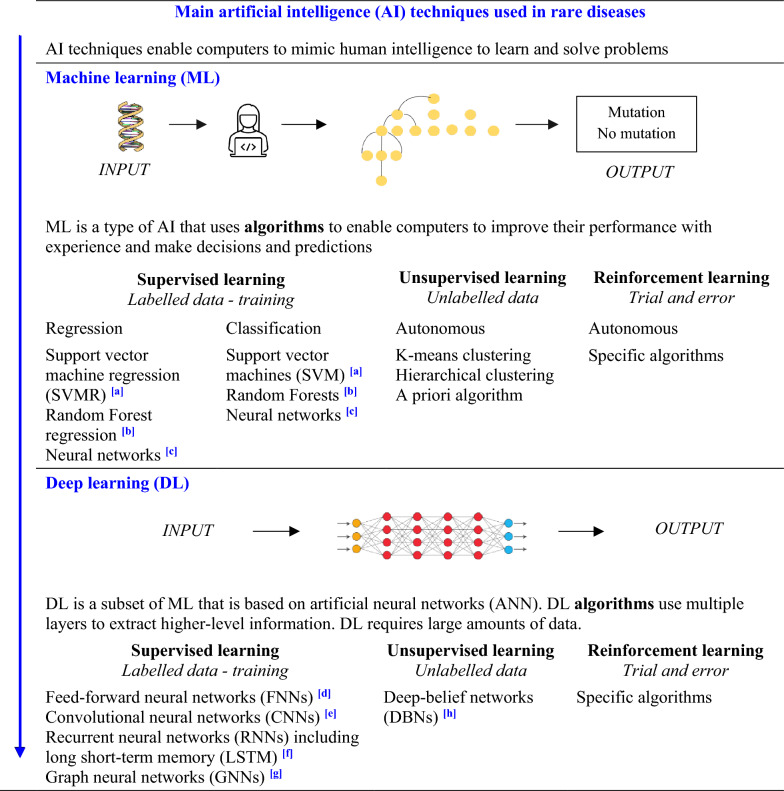
Overview of artificial intelligence techniques in rare diseases

AI, artificial intelligence; ANN, artificial neural network; CNN, convolutional neural network; DBN, deep-belief network; DL, deep learning; FNN, feed-forward neural network; GNN, graph neural network; ML, machine learning; RNN, recurrent neural network; SVM, support vector machine. The arrow on the left indicates the increase in the amount of data that can be processed and the greater accuracy of the results

a. SVM can be used for text or image classification, gene expression analysis, facial analysis, and anomaly detection

b. Random Forest combines multiple decision trees to predict the output. It can be used for regression and classification to predict numerical variables or categories

c. Neural networks are particularly useful for image classification and facial analysis

d. FNNs involve data that travel in the forward direction only from the input to the output layer. They can be used for pattern recognition

e. CNNs involve weighted connections between artificial neurons or units that will determine the impact of one unit on the other. CNNs can be used in computer vision, speech recognition, and image processing

f. RNNs involve data that travel bidirectionally. They memorize the output of a layer and then use this memory to improve the output of the next layer. They can be used in speech to text conversion

g. GNNs involve graphical data. They are widely used in computer vision for image classification and can establish spatial relationships between the objects within an image. They are used in natural language processing to form relationships between words and phrases. GNNs can create models that make accurate predictions

h. DBNs involve multiple layers of artificial neurons or units, namely visible and hidden layers. They can learn hierarchical data representation

Adapted from [2, 3, 13, 15, 17, 18]

Deep learning (DL) is a sub-category of supervised learning that can be applied to unsupervised and reinforcement methods. DL approaches are used for complex problem-solving and large-scale data analysis (e.g., image and textual analyses). DL is based on artificial neural networks (ANN) that are made up of artificial neurons arranged in a series of layers, through which information is passed on from one layer to another. DL algorithms can learn to recognize complex patterns and relationships in large amounts of data and are particularly performant for computer vision (e.g., image classification and segmentation), natural language processing (NLP; automatic text generation and identification, language translation, speech recognition, etc.) and generative AI (text and images) (see Table 1).

Several types of ML, including DL methods, can be combined to achieve a multimodal approach. As each method has its own specificities for processing different types of data, such as images, text, and tables (see Table 1), this multimodal approach produces more accurate results. Choosing the right method or algorithm thus requires advice from an AI expert.

### How can AI technologies be of benefit to rare diseases?

In the European Union (EU), a disease is defined as rare if it affects ≤ 1/2000 persons [19], whereas in the United States US) the term “rare” refers to a disease or condition that affects less than 200,000 persons [20]. As about 300 million people worldwide are affected by a rare disease, these conditions represent a considerable public health burden [21]. More than 10,000 rare diseases have been identified so far, which makes physician training and patient care challenging [21–23].

For patients with rare diseases, the period between the appearance of the first symptoms and the correct diagnosis is often long [22]. The main reasons for this delay, known as a “diagnostic odyssey”, are a lack of physician awareness about rare diseases, and the unspecific clinical profile of many of these conditions. In addition, one given syndrome may be associated with several genes (genetic heterogeneity), and, inversely, two clinically different syndromes may be due to variants in the same gene (allelic series), possibly delaying the diagnosis. During this period of diagnostic wandering, which is often compounded by a lack of specific diagnostic tools [15], two-thirds of patients may experience misdiagnosis and receive ineffective or inappropriate treatments, whereas other patients may remain in a state of “diagnostic impasse”. The longer the odyssey, the higher the risk of disease progression and health impairment, especially for the ~ 5% of rare diseases for which there is an effective and specific treatment. For example, patients with Fabry disease can be treated either with enzyme replacement therapy (ERT; available since 2001) [24, 25] or, in case of amenable pathogenic galactosidase alpha (*GLA*) gene variants, with a pharmacological chaperone therapy (available since 2016) [26, 27]. When initiated early enough, both types of therapy prevent or stabilize the progressive organ damage that occurs in patients with this disease [28, 29]. Accelerating diagnosis is therefore a key step for slowing down the progression or stabilizing the clinical signs and symptoms of rare diseases. In addition, the accurate and early diagnosis of a genetic rare disease may not only benefit the index case but the whole family [22] as it is estimated that 80% of rare diseases are inherited, of which at least 70% have onset during childhood [30]. Machine-based analysis of large amounts of data using AI techniques may help physicians make an earlier and more accurate diagnosis, predict the progression of the disease, and when available, identify the most appropriate treatment strategy to ensure long-term effectiveness and maintain treatment adherence [15].

Furthermore, combining AI methods is of particular interest for the differential diagnosis of rare diseases, particularly in case of clinical overlap or similarities with another rare disease, a more common disease, or a physiological process (e.g., Fabry disease and multiple sclerosis, or Fabry disease and ageing) [31].

The rapid increase in research into the application of AI to rare diseases has been accompanied by the publication of numerous recent reviews highlighting general advances in the field [1–11]. In this narrative review, we present some of the latest applications of AI that have been developed or adapted to facilitate and accelerate rare disease diagnosis, prognosis, and tailored treatment, with a structured review of the literature to highlight lessons learnt from Fabry disease.

## Literature search methodology

Literature searches were conducted using double and triple combinations of keywords related to AI and Fabry disease (AI and Fabry disease, AI and rare diseases and Fabry disease, ML and Fabry disease, DL and Fabry disease) to search the Medline database via the PubMed online search engine. The end date for the literature search was 08 January 2025. No specific filters were applied. Results of each search were merged, and duplicates were eliminated. Among 23 retrieved articles, 8 were excluded (5 were out of scope, 1 presented only preliminary data, and 2 were out of date). The list of 15 specific articles related to AI and Fabry disease was supplemented by articles identified through manual searches using double combinations of keywords related to AI and each organ affected in Fabry disease, and by articles containing a broader view of the subject identified through searches using double combinations of keywords for AI and rare diseases (limited to articles published between 01 January 2021 and 31 December 2023). The list of 20 articles included in the structured review of the use of AI in Fabry disease is provided in Table 2.

**Table 2:** List of articles selected for the structured review of artificial intelligence and Fabry disease

Differential diagnostic	Issue resolved by artificial intelligence for patients with Fabry disease	Algorithm type	References
Global	Screening for patients with Fabry disease from EHRs	ML NLP-based approach	[32, 33]
Transcriptomic and proteomic profile	Differentiating patient transcriptomic, metabolic and proteomic profiles	ML	[34–38]
Craniofacial dysmorphology	Facial recognition with frontal photographs	DeepGestalt ML algorithm	[38–41]
Cardiomyopathy	Detection and distinction of cardiomyopathies and other diseases including Fabry disease based on electrocardiogram (ECG) or cardiovascular magnetic resonance imaging (cMRI)	Text mining ML DL	[42–45] [46] [47, 48]*[49]
Brain lesions	Differential diagnosis between Fabry disease and brain ageing	DL	[31]
Renal disease progression	Kidney disease progression through assessment of podocyte foot process width	DL	[50]

cMRI, cardiovascular magnetic resonance imaging; DL, deep learning; ECG, electrocardiogram; EHR, electronic health record; ML, machine learning; NLP, natural language processing; (*), reviews

## Applications of artificial intelligence to rare diseases

The spread of AI in the field of rare diseases illustrates, more broadly, the challenges of the current technological revolution. Its application is not homogeneous across therapeutic areas and uses technological tools with differing rates of development. However, the overall direction of movement is marked by a very powerful acceleration, and the field of rare diseases constitutes one of the sectors in which these advances are most visible in healthcare.

AI is mostly used to facilitate rare disease diagnosis, either using a prospective approach to identify patients possibly affected by a rare disease in large populations or using a retrospective approach to diagnose a previously overlooked rare disease. In this review, we concentrate on methods and approaches that are relevant to Fabry disease, although these methods may also be applied to any rare disease affecting multiple organs (see Table 3).

**Table 3:** Overview of available algorithms recently developed and applied to rare diseases

Rare disease issues	Aim and type of artificial intelligence, machine learning, deep learning methods and algorithms		References
Identification of potential rare disease patients within a large population of subjects or patients			
Prospective approach			
Screen newborn genome	Screen4Care to accelerate diagnosis and flag patients at risk of rare disease	ML	[53]
Screen patient EHR	Phenotypic risk score	ML/NLP	[54, 55]
	Screening for Fabry disease patients from EHRs	ML/NLP	[32, 33]
Clinical records	Detection of abnormal phenotypes	LLM/CNLP	[56, 57]
Patient-centred approach – Sequential and combined AI techniques			
Retrospective approach			
Genomic approaches	Prioritization and nomination of variants Prioritization of missense variant pathogenicity	CNLP DL	[58, 59]
Transcriptomic and proteomic approaches	Differentiating patient transcriptomic, metabolic and proteomic profiles	ML	[34–38]
Craniofacial dysmorphology	Facial recognition with frontal photographs External ear shape morphology Frontal and lateral facial photographs taking into account the outline of the cranial vault, and the position and morphology of the external ear	ML CNN	[39–41, 60, 61]
ECG signature	Differential diagnosis of Fabry disease		[48, 62, 63]
Cardiovascular Magnetic Resonance imaging (cMRI)	Detection and distinction of cardiomyopathies and other diseases including Fabry disease	ML Text mining DL	[42–45, 49]
Brain lesions	Differential diagnosis between neurological diseases or between Fabry disease and brain ageing	DL	[64, 31]
Retinal fundus profile	Differential common or rare disease diagnosis Sex classification	DL	[65–68]
Hearing impairment	Self-audiometry testing Tinnitus functional index	DL	[69–71]
Skin phenotypic signature	Differential diagnosis	DL	[72]
Renal signature	Kidney disease progression through assessment of podocyte foot process width	DL	[50]
Multimodal approach	All available data for a patient	NA	[73]

cMRI, cardiovascular magnetic resonance imaging; CNLP, clinical natural language processing; CNN, convolutional neural network; DL, deep learning; ECG, electrocardiogram; EHR, electronic health record; LLM, large language models; ML, machine learning; NA, not applicable

Fabry disease (Online Mendelian Inheritance in Men® OMIM #301,500) is a rare, progressive, X-linked, multisystemic, lysosomal disorder caused by pathogenic genetic variations in the *GLA* gene, which encodes α-galactosidase A (α-Gal A) [51]. A large variety of *GLA* variants underlying the phenotypic heterogeneity of the disease have been identified, and over 1000 different *GLA* variants have been reported so far. Most variants are ‘private’ and confined to individual pedigrees with possible variability in phenotypic expression resulting from phenotype-modifying factors (e.g., genetic background, epigenetics, and environmental conditions) [52]. Quantitative or functional α-Gal A deficiency leads to the accumulation of undegraded globotriaosylceramide (Gb_3_) and its deacylated derivative globotriaosylsphingosine (Lyso-Gb_3_) in body fluids and in a variety of tissues [52]. This causes progressive damage to the affected organs, life-threatening complications, and an increased risk of premature death. Fabry disease can be classified into classic and non-classic (later-onset) phenotypes [29]. Male patients with classic Fabry disease have severely reduced or no residual α-Gal A activity and generally experience signs and symptoms such as neuropathic pain, cornea verticillata, gastrointestinal symptoms, hypohidrosis, and angiokeratoma from childhood. Long-term disease manifestations include proteinuric chronic kidney disease leading to renal failure, hypertrophic cardiomyopathy, cardiac arrhythmia, hypoacusia, and stroke [29]. Later-onset Fabry disease, a milder phenotype, is seen in patients with residual α-Gal A activity. Early clinical features are usually absent and disease manifestations associated with key target organs may be absent or mild, occur later in life, and be limited to cardiac involvement, which can be severe. The severity of the disease is more variable in females because of X-chromosome inactivation [29].

### Prospective approaches

#### Identifying candidates at risk of a rare disease in a large population of infants by targeted sequencing

As rare diseases mostly affect children and treatment efficacy may be time-dependent, identifying a rare disease as early as possible may have a crucial impact on the patient’s health. Screen4Care (S4C, [74] is a public health programme that focuses on treatable and actionable rare diseases and aims to accelerate rare disease diagnosis by focusing on two central pillars: screening a large European population of newborns (~ 25,000) for rare diseases of genetic origin with next-generation sequencing (NGS), and using digital technologies to generate predictive algorithms and flag patients at risk of specific rare diseases through electronic health record (EHR) screening. Diagnosed patients may be treated and followed up to enrich knowledge of the natural history of their disease, creating new information that will be used to feed ML algorithms in a virtuous circle [53]. However, the ethical issues surrounding neonatal diagnosis remain a matter of debate, especially for late-onset rare diseases, such as the later-onset form of Fabry disease, as this information may change the behaviour of parents and constitutes a heavy burden for the patient. Postponing screening for late-onset diseases until early adulthood could be a compromise. In contrast, patients with the classic form of Fabry disease would benefit from neonatal diagnosis and early treatment initiation, as illustrated by the better outcomes reported for (asymptomatic) ERT-treated male patients aged seven to eight years old [75, 76].

#### Identifying candidates at risk of a rare disease in a large population of patients by screening hospital electronic health records

Hospital EHRs constitute a precious and wide source of information that can be used to develop and evaluate ML-based screening and NLP methods to identify rare diseases through a two-step process. First, phenotypes and clinical signs are extracted from all EHRs in a phase called “name entity recognition” (NER). This phase involves parsing unstructured narratives in clinical notes to detect terms related to healthcare, such as symptoms, diagnoses, or treatments. NER requires manually annotated documents to learn to recognize the relevant concepts to extract. Then, the identified words and expressions are normalized by aligning them to a standardized vocabulary, such as the Human Phenotype Ontology (HPO) [77, 78] or SNOMED-clinical terminology (SNOMED-CT) [79], ensuring consistency and interoperability across systems. In addition, NLP detects the context associated with the extracted medical concepts, for example, the certainty (assertion, negation, or suspicion) and the experiencer (i.e., if the concept is related to the patient or their family history) [80]. Second, obtained data are processed to calculate phenotypic distances, either between each patient and disease using a knowledge database [54], or between each patient and a patient with a validated diagnosis [55]. The files of patients with top-ranked scores are then examined by rare disease expert physicians who decide whether or not to perform complementary tests to confirm the diagnosis. This NLP-based approach is of particular interest for rare metabolic diseases that affect multiple organs and induce a variety of clinical signs and symptoms, and thus often require several hospital visits before accurate diagnosis. Two groups, one from the USA [32] and one from Poland [33], have developed ML methods to support physicians in detecting undiagnosed patients with Fabry disease. This NLP-based approach to diagnosis emphasizes the need for maintaining accurate EHRs, a process that may be facilitated by the use of voice recognition AI technologies to extract relevant information from patient and physician discussions during consultations.

Furthermore, the emergence of large language models (LLMs), such as ChatGPT, LLAMA, BLOOM, and VICUNA, opens new perspectives for the reuse of textual data that remain largely unexplored [56]. These LLMs are advanced AI systems trained on extensive text corpora to understand and generate human-like language. They can process vast amounts of literature and clinical data and are particularly performant for identifying patterns and extracting relevant information from unstructured medical texts. Recently, the LLM approach has been used to enable the detection of abnormal phenotypes in clinical records and published case studies [56, 57].

### Retrospective patient-centred approaches

#### Identifying pathogenic genetic variants using genome sequencing and AI-based variant prioritization tools

As rare diseases are mostly of genetic origin, the genome of patients with a suspected rare disease may be searched for genetic variants. NGS is a massively parallel DNA sequencing technique that enables billions of DNA regions/fragments to be sequenced in parallel with high accuracy. Alongside the use of panels of specific genes or genomic regions (“targeted NGS”), NGS can be used to sequence all the exons of the ≅ 20,000 protein-coding genes representing 1–2% of the entire genome (whole-exome sequencing or WES), or the whole genome (whole-genome sequencing or WGS). WES sequencing is used to identify variants associated with very heterogenous disorders [81], whereas WGS is used to detect mutations in enhancer regions and search for structural variants such as deletions, duplications, and copy number variants (CNVs), or in case of failure of WES [82]. Both WES and WGS generate large amounts of genomic data that, despite the emergence of several AI-based tools to detect and prioritize variants, still require extensive and time-consuming analysis by specialists in bioinformatics to identify disease-causing variants among the large amounts of non-pathogenic genetic variations [83]. To help overcome this limitation, De La Vega et al. [58] developed an AI-based method to make genomic data interpretation easier for non-expert physicians and save time. The method of prioritization used in the study by De La Vega et al. was shown to perform better than similar tools for matching single nucleotide variants (SNVs) or structural variants extracted by WES or WGS. The prioritization method was also combined with patient phenotypes extracted from clinical notes manually or by clinical natural language processing (CNLP). The output achieved by combining the prioritization method with deep CNLP phenotyping includes automated diagnosis nomination. The pathogenicity of Fabry disease variants could also be evaluated by a supervised DL algorithm. This algorithm has already been shown to be effective at prioritizing missense variant pathogenicity (MVP) for congenital heart diseases and autism spectrum disorders [59]. Nonetheless, expert clinical evaluation remains necessary to validate the final diagnosis. Large-scale omic-based approaches have also been extended to analysis of the proteome, as illustrated by studies conducted with next-generation plasma proteomics in Fabry disease patients. Combined system biology and ML-based approaches have already been shown to allow the transcriptomic [37], metabolic [34] or proteomic [35, 36, 38] profiles of patients with Fabry disease to be differentiated from those of patients with other diseases and controls. Correlations between genomic and proteomic profiles in metabolic diseases could help in the development of personalized therapies.

#### Identifying patients with rare diseases by analysis of their clinical profile using AI-based tools

##### Facial morphological signatures

Up to 40% of the 7000 identified rare diseases may lead to dental, oral, or craniofacial anomalies [84], the diversity of which is a major obstacle to rapid diagnosis for non-expert physicians. Powerful AI-based methods of facial pattern recognition can be used as valuable tools to support the differential diagnosis of one rare disease from thousands of syndromes, with tools such as DeepGestalt already being developed and containing a database of frontal facial photographs associated with genomic and phenotypic information [39]. Automated facial recognition techniques have been shown to provide results that are consistent with or more accurate than human assessments, highlighting their usefulness in common medical practice [85]. Some Fabry disease patients present subtle facial particularities [86] that could be diagnosed by running DeepGestalt [41], therefore supporting the idea that such tools may be of benefit for other rare diseases with subtle facial phenotypes. The more these ML-based facial recognition methods are used, the better they will be able to perform. The feeding of new information will also make them more universal, particularly in terms of the age and ethnic origin of the patients [40]. These improvements rely on the willingness of patients or their representatives to consent to the sharing of their facial photographs [85]. This is a very significant example of the importance of obtaining patient consent when collecting sensitive data. New ML algorithms for facial recognition have been set to either combine frontal and lateral facial pictures [61] or to focus on the morphology of the external ear [60] for the diagnosis of specific rare diseases. The high level of accuracy of these techniques has already been demonstrated by their ability to distinguish between Kabuki syndrome variants [61].

##### Cardiac functional and morphological signatures

Impaired heart activity and morphology can be detected by non-invasive medical investigations like electrocardiograms (ECGs) and cardiac imaging, respectively. However, given the large number of cardiomyopathies, accurate interpretation of the test results is a complex task, which can be facilitated by AI-based tools. The early detection of cardiac symptoms in patients with Fabry disease is crucial, as organ damage can mostly not be reversed, but only slowed down, by available therapies [87]. Nine independent ECG parameters were recently identified as specific and accurate predictors of Fabry disease in subjects not yet presenting lateral ventricular hypertrophy [88]. In addition, for symptomatic patients, five ECG parameters have been integrated into a multiparametric ECG score-based algorithm to facilitate the differential diagnosis of Fabry disease versus other hypertrophic cardiomyopathies [48, 62]. The longitudinal comparative analysis of ECG traces from patients with Fabry disease and healthy subjects during adulthood revealed that the rate of change of ECG parameters over time was a hallmark of the disease that showed male and female specificities, and thereby constituted a potential reference for monitoring disease progression [63]. Altogether these studies illustrate how knowledge of disease-specific ECG trace characteristics could be used to feed ML algorithms to screen, manage, and follow-up patients with a specific rare genetic condition.

The combination of ECG and ML approaches with cardiovascular magnetic resonance imaging (cMRI) could further improve the detection of and distinction between cardiomyopathies, as already observed with ECGs and echocardiograms trained on federated learning models across multiple institutions [42]. Another study has indicated that three-dimensional myocardial deformation analysis (3D-MDA) could be used for training ML-based cardiomyopathy diagnostic tools [46]. The 3D-MDA used in this study relied on architectural and deformation data (wall thickness, strain amplitude, and time to peak-systolic strain) generated by cMRI conducted on 163 patients with cardiomyopathy [46]. A feed-forward neural network model trained on 900 phenotypic features from 3D-MDA was shown to perform better than threshold-based measures of volumetric or strain-based markers (AUC 0.94 vs. 0.70 or 0.64, respectively) for the classification of hypertrophic cardiomyopathy versus disease phenocopy states, such as Fabry disease, cardiac amyloidosis, and hypertensive cardiomyopathy. In addition, a study using a preliminary ML-based method to measure the thickness of the left ventricular wall on cMRI scans demonstrated that the ML-based analysis showed higher precision than an international panel of 11 cardiovascular imaging experts (8% versus 20% of the patients were assigned an alternative diagnosis compared to their actual diagnosis by the ML-based method and experts, respectively) [43]. This method involved a first step of deep fully convolutional neural networks (CNNs) for ventricular segmentation, trained on left ventricular short-axis images (N = 1439) from 60 patients with hypertrophic cardiomyopathy, and then a quality control step performed by humans. Although the method remains to be fully validated, it is expected to improve the diagnosis of clinical hypertrophic cardiomyopathy versus subclinical hypertrophic cardiomyopathies found in other pathologies such as hypertension, Fabry disease, amyloidosis, and aortic stenosis. It will also likely prove useful for the selection of Fabry disease patients for clinical trials evaluating new therapeutic options. A 3D CNN model trained on MRI left ventricular short-axis images without segmentation has also been developed to distinguish between hypertrophic cardiomyopathy and Fabry disease [49]. This model, referred to as the short-axis view left ventricular hypertrophy classifier, has been tested in a small, single-blind, external validation study (N = 20 patients with Fabry disease and N = 11 with hypertrophic cardiomyopathy) with promising results (*F1-*score: 0.727; accuracy: 0.806; and AUC: 0.918) suggesting that this model could be a potentially valuable tool for differential diagnosis. ML has also been applied to allow the automated evaluation of cardiac structure and function on images obtained by conventional cMRI. Davies et al. [44] developed an algorithm measuring left ventricular (LV) mass and global systolic function using the left ventricular ejection fraction (LVEF). This CNN-based algorithm was trained on 1923 scans and its performance was validated for precision and generalizability by analysing two cohorts of patients with multiple diseases (N = 109 and N = 1277, respectively). The algorithm performed faster than three human experts (20 s vs. 13 min) and was also more precise, as indicated by significantly lower scan-rescan coefficients of variation (*p* < 0.05) for LV mass (3.6% for the algorithm vs. 4.8% for the experts) and LVEF (4.2% for the algorithm vs. 6.0% for the experts). LVEF is used to detect myocardial damage in clinical practice and as a surrogate endpoint for drug development. This ML algorithm for cardiac volumetric analysis on cMRI scans may therefore facilitate clinical decision making and allow the size of patient cohorts in clinical research studies using LVEF as an endpoint to be reduced by 46%.

Finally, AI tools have also been investigated as a way to identify patients with unexplained LV hypertrophy via automated reading of EHRs. In the study by Sammani et al. [45], data from 26,954 patients were used for training, including 204 cases (0.8%) with undiagnosed lateral ventricular hypertrophy (56 related to amyloidosis and two related to Fabry disease). A text-mining algorithm using clinical discharge letters and records, and an ML-based algorithm trained on echocardiographic images of patients with diagnosed LV hypertrophy were tested [45]. Both methods were able to flag potential cardiac hypertrophy cases with high specificity (0.99 for ML vs. 0.67 for text mining), but the sensitivity of the ML method based on echocardiographic images was lower than that of the text-mining algorithm (0.32 vs. 0.78, respectively).

##### Brain lesion phenotypic signatures

Brain lesions, analysed by magnetic resonance imaging (MRI), may be indicative of both rare and more common diseases. For example, progressive brain white matter lesions can result from multiple sclerosis, Fabry disease, or ageing. An AI-based system was recently developed to enhance MRI differential diagnosis [64]. Lesions were first detected by a DL-based CNN algorithm using fluid-attenuated inversion-recovery (FLAIR) sequences, then characterized through image processing and combined with expert knowledge to generate a disease probability score through Bayesian inference. The system was trained (N = 86 studies) and tested (N = 92 studies) on images encompassing 19 diseases, including six rare diseases. Overall, the performance of the AI-based system was similar to that of radiologists for differential disease diagnosis, and the AI-based system performed almost as well as academic neuroradiologists for the specific diagnosis of rare diseases. This approach could help raise awareness of rare diseases in first-tier imaging centres.

To overcome the lack of neuroimaging biomarkers and determine whether the brain of patients with Fabry disease is prone to accelerated ageing, Montella et al. [31] developed and validated a DL brain-age model, which was trained on T1-weighted brain scans (N = 2160, 8 public resources) of healthy subjects (mean age of 33 years, range 3 to 84 years) and based on DenseNet architecture for reproducibility. When applied to an internal cohort of 52 patients with Fabry disease (mean age of 40.6 years, and median Fabry stabilization index—FASTEX—score of 6) and healthy subjects (mean age of 38 years), the brain-age model predicted that the brain age of patients with Fabry disease was higher than that of the healthy subjects (estimated marginal means: 3.1 vs. − 0.1, *p* = 0.01), In addition, greater brain-predicted age difference (brain-PAD) values for age and sex in patients with Fabry disease were found to be associated with FASTEX scores, the brain parenchymal fraction, white matter hyperintensity loads, and tissue volume reduction throughout the brain. These data suggested that the progression of Fabry disease might be influenced by alterations in brain age, and that brain-PAD values generated by the DL brain-age model could be used in clinical monitoring and drug development.

##### Eye phenotypic signatures

Ocular imaging may reveal the presence of diseases at their earliest stages and thus constitutes a powerful diagnostic tool that may, in some cases, be amenable to telemedicine. As for brain MR images, DL-based CNN algorithms have been used for retinal or macular segmentation, allowing the spatial structure of the images to be preserved and enabling highly sensitive and specific distinctions to be made between phenotypes, as demonstrated for several pathologies including diabetic retinopathy [65]. Most patients with classic Fabry disease develop ophthalmic features in all segments of the eyes, including the retina, for which ML-based automated detection could be developed and integrated alongside other evaluation tools to accelerate diagnosis. For example, two rare diseases with retinal abnormalities—recessive Stargardt disease (STGD1), which leads to early retinal degeneration and vision loss, and Moyamoya disease, which may affect the blood vessels of the eye leading to several ocular symptoms—can be diagnosed through fundoscopic examination. An ML-based algorithm has been shown to be able to predict spatially-resolved retinal function or “inferred sensitivity” in patients with STGD1 by analysing the results of fundus-controlled perimetry (FCP or microperimetry) visual field tests [66]. In addition, a DL-based algorithm has been developed to screen and stage patients with Moyamoya disease by analysing vessels in photographs of the retinal fundus [67]. Both studies aim to use these tools as biomarkers during clinical research for the development of new drugs. Moreover, a DL-based algorithm trained on a large dataset (84,743 photographs from the United Kingdom Biobank) has been shown to be able to classify the sex of healthy subjects with high specificity (83.6%), precision (87.3%) and accuracy (86.5%) based on analysis of retinal fundus photographs (N = 252) [68]. When challenged with an external dataset including images from patients with a foveal pathology, the performance of the method declined, thus pinpointing the importance of the fovea region in determining between-sex differences in retinal features.

##### Hearing loss and tinnitus phenotypic signatures

Several ML-based methods have been developed for automated hearing loss evaluation and sensorineural prediction, including self-audiometry testing that can be conducted with a smartphone [69, 70]. Recently, a deep neural network was developed to predict tinnitus treatment outcomes by analysing electroencephalogram (EEG) data that were represented and interpreted as images using a CNN algorithm [71]. The ease and general availability of hearing monitoring supports its wide implementation as a method to identify diseases, and particularly to facilitate differential diagnosis. Hearing loss is a frequent symptom of Fabry disease and significantly impairs patient quality of life [89]. Its automated detection, in parallel with EEG analysis, may contribute to earlier diagnosis. In addition, the AI-based tinnitus functional index may potentially be used to evaluate disease progression or the effectiveness of specific therapies for this symptom.

##### Skin phenotypic signatures

A large number of rare diseases with cutaneous involvement have been identified (N > 800). An AI-based technique has been developed to assist skin disease diagnosis and could be further elaborated for analysis of the dermatological manifestations of some rare diseases. Using a four-layer CNN model (4-CNN), the AI approach was shown to be able to classify seven types of pigmented lesions from 10,015 dermoscopy images collected from patient populations with various skin types and diagnoses confirmed using different methods [72]. Although the diversity of the dataset needs to be improved, the 4-CNN method showed high average levels of accuracy (93.6%), sensitivity (95.9%), and specificity (98.3%) for lesion diagnosis. As previously mentioned, some Fabry disease patients develop vascular cutaneous lesions (angiokeratomas) that are generally located in the genital areas or on the upper thighs, lower back, and buttocks, and less frequently elsewhere on the body [90]. Like ophthalmic and hearing monitoring, dermatological evaluations could be integrated into other ML-based diagnostic methods for the diagnosis of rare diseases with cutaneous involvement, including Fabry disease, with further improvements being anticipated as the capacity for assessing increased pixel numbers increases.

##### Rare disease prognosis and patient-tailored treatment

Physicians rely on knowledge of the natural history of a given rare disease for patient prognosis and to make informed therapeutic choices when treatments are available. AI-based models integrating large amounts of data may provide additional support to physicians in these cases. Indeed, a quantitative retrospective natural history modelling (QUARNAM) method has already been developed for gathering all available information, including data from case reports, on seven rare neurogenetic disorders [91]. Monitoring disease progression in treated patients is of utmost importance for evaluating therapy effectiveness and adapting treatment strategies when required [3]. An example of how AI can be used to monitor disease progression is provided by developments in the assessment of kidney disease. One way to follow kidney disease progression is to assess podocyte injury by measuring the increase in variability of podocyte foot process width (FPW) that is associated with many kidney diseases. A network architecture was created to capture the podocyte-glomerular basement membrane interface (PGMBI) and the foot process slits, which were semantically segmented to train a CNN-based algorithm [50]. When applied to electron microscopy images of kidney biopsies from patients with Fabry disease (N = 56), type 2 diabetes (N = 15) and minimal change disease (N = 10), as well as from healthy subjects (N = 17), the CNN method produced accurate measures of FPW that significantly correlated with unbiased stereology data generated by expert technicians (r = 0.92, *p* < 0.0001). Notably, the data were produced much faster using the DL method (< 1 min vs. several minutes). This DL-based method for assessing podocyte injury may therefore be used to predict disease progression and help inform treatment choices for patients with rare diseases associated with kidney disease.

## Ongoing benefits, challenges, limitations, and future perspectives for the use of artificial intelligence in the field of rare diseases

**Fig. 1:**
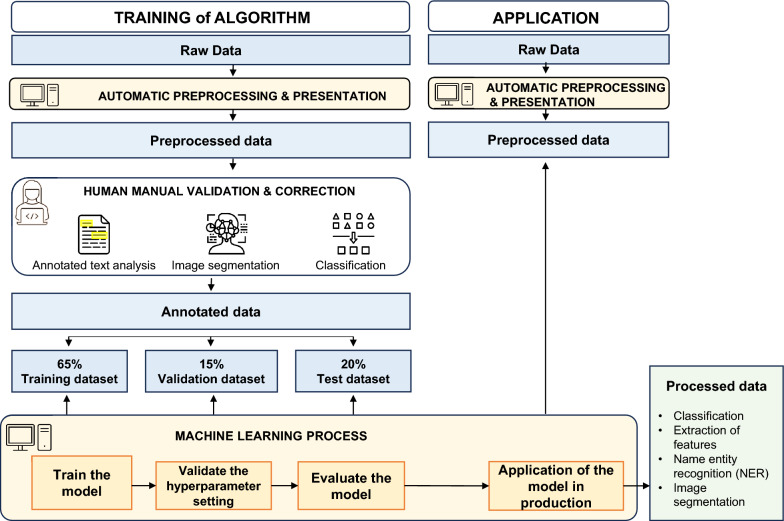
Scheme of the machine learning common process combining human input and computer activities. The algorithm is first trained with a set of raw data that are pre-processed automatically and presented. Generated pre-processed data are validated and corrected manually by an AI expert. These annotated data are distributed for use as the training dataset (65%), for validation (15%) and to test the machine learning process (20%) that trains the model, validates the hyperparameter settings, and evaluates and applies the model in production This process generates processed data such as classified images, extracted features, recognized name entities, and segmented images. The model is then applied in production to other sets of automatically pre-processed data

Rare genetic diseases are often complex, particularly when they affect multiple organs and are associated with symptoms that can be observed in common diseases. This makes the diagnosis difficult to achieve, especially for non-expert physicians facing a scarcity of human resources. The analysis of each symptom separately may lead to misdiagnosis, whereas an accurate diagnosis is more likely to be achieved when the symptoms are considered as a whole. This process can be facilitated by AI-based techniques that enable the evaluation and integration of large amounts of various types of data. For AI methods to be applied in this context, a common process combining human and computer activities is required for the collection, manual annotation, extraction and normalization of the data, before training can be initiated on an appropriate data set and then further validated before application to a test data set (see Fig. 1). Overall, AI-based techniques have been found to be equivalent to, or better than, experts for assessing some diagnoses [43, 64, 85], likely because, in contrast to machine-driven analyses, physicians are prone to subjectivity in data interpretation and may rely solely on their knowledge rather than on the wide range of available resources (e.g., OMIM [92], Orphanet [93], and Gene reviews [94]). In a comparative analysis of the evaluation of frontal facial photographs of patients, medical geneticists and AI-based tools were found to focus on different image features, thereby suggesting that they could learn from each other to further improve their performance [95]. The integration of several AI models in a multimodal ML model [73]—combining EHRs, laboratory data, WES/WGS results, and imaging—could also potentialize the accuracy of each AI model used independently. Although such multimodal approaches pave the way for improvements in diagnosis, it should be noted that the results generated by AI are not final and have to be validated by experts who will make decisions for optimizing patient management based on consideration of the patient “as a whole”. Predictive computational models have led to the emergence of the medical digital twin concept, which may contribute to personalized medicine by helping to find or adapt specific treatments for patients with rare diseases [96]. For example, data collected in real-time for a patient on treatment can be used by ML-trained algorithms to simulate the effect of the drug in an untreated patient and predict outcomes. In addition, in silico medicine technologies using virtual patients to generate placebo controls may compensate for the small number of eligible patients with rare diseases in clinical trials. When algorithm training is possible with small amounts of data, these methods may allow adjustments to dose regimens to be made as part of a personalized medicine approach. Beyond accurate diagnosis and finding treatments, AI-based techniques may be integrated into wearable biosensors and non-invasive devices (e.g., rings, headbands, and contact lenses) for the remote monitoring of patient physiological metrics (e.g., vital signs, cardiac function, EEGs, and intraocular pressure), with the output being used to trigger specific healthcare responses when necessary as part of “hospital-at-home” initiatives [97], or for patient-reported outcomes (PRO) in patients enrolled in clinical trials [98].

### What are the challenges and limitations of the use of AI for rare diseases?

In line with its development in healthcare over the past 10 years, AI faces several challenges and limitations. AI-based models are trained and tested through methodologies that rely on well-designed data sets to optimize performance independently of patient age, sex, and ethnicity [40]. Modifying data set training has been found to increase diagnostic yield from 57 to 82% [39], illustrating the essential role played by AI experts in selecting and setting up the AI tool. In addition, the starting material should ideally be based on large training sets containing data that are heterogenous enough to represent all types of patients [40] and mitigate the risk of biased estimates and silent failures, but homogenous enough to be used across different healthcare institutions [42] or in multimodal approaches [73].

The application of AI technologies to the management of rare diseases is often hindered by the scarcity of data. Various techniques may help mitigate this issue such as the use of region-based CNNs to augment image data by modifying photograph angles by 10 °C  , the use of Bayesian CNNs that have relatively high accuracy and performance, and the introduction of hybrid algorithms that integrate rule mining and AI [5]. However, it should be noted that for conditions associated with very typical facial dysmorphism (e.g., progeria syndrome), even small amounts of data have been found to be sufficient for highly accurate AI detection of known phenotypes, and in these cases AI may also contribute to the identification of new ultra-rare phenotypes [99].

Beyond the lack of external evaluation [17, 100], limitations on the use of AI in healthcare relate to explainability [101], including how to manage the opacity of AI methods or algorithms for both healthcare practitioners and patients, how to assess and adjust to the reliability and reproducibility of the models, and how to interpret the results. Improving explainability will not only increase physician and patient confidence in these tools but will also allow more robust evaluation of the trustworthiness of the AI methods and algorithms. Hence, developers may prefer models with transparent values that allow physicians to fully understand how the model handled the data and achieved the outcomes [43]. The Precise4Q consortium raised the issue of the importance of having a multidisciplinary perspective for explainability—involving AI designers, healthcare professionals and legislators—to prevent the misuse of AI in public health [101].

Despite the plethora of research publications demonstrating the usefulness and high performance of AI-based models in the management of health data for rare diseases, only a few projects are currently undergoing further processing for general application. This likely results from the elevated cost of implementing new technologies, alongside the fear that rapid advances in the field could render the investment outdated within months, as well as practical issues such as the lack of continuum between medical records from general practice and hospitals during the patient journey preventing the constitution of associations in mega databases. Nonetheless, by facilitating clinical decisions, providing predictions and reducing the sample size required for clinical trials, AI-based systems should ultimately result in cost savings, particularly in areas such as the future development of new therapeutics [102]. In France, current AI-based projects that have reached production status mainly focus on image recognition and classification, and on the optimization of healthcare pathways for patients with type-1 diabetes (Diabeloop) [103] or those taking an oral anti-cancer drug at home (Resilience care [104]). In addition, the use of ML-based algorithms for rare disease diagnosis, which were developed within the Necker hospital and leverage EHRs and NLP, has started to spread across French hospitals [105, 106]. Standards that allow for the interoperability of algorithms between countries also need to be anticipated [107], especially for patients with rare diseases who likely participate in international clinical trials.

### How can patients be protected by human oversight of AI?

The drawbacks of easy access to large health data sets relate to major concerns over patient privacy, safety and protection from AI deviations [108]. After several years of discussion, the European directive text about AI—the “AI act”—was agreed on 08 December 2023, finalized on 22 January 2024, and will be applied gradually over 6, 12, 24 and 36 months. The “AI Act” establishes harmonized rules for AI designers and users among EU countries while preserving AI innovation, and thus makes the EU a pioneer in this domain. Four levels of risk have been established: unacceptable, high risk, limited risk, and minimal risk, with specific rules for high-risk innovations. The “AI Act” also includes the concept of “human oversight”, which was first proposed by Ethik-IA [109–111] within the framework of the re-examination of the 2021 French Bioethics Law. The human oversight principle involves human control of AI from design to use, ensuring competency, formation, authority, and appropriate resources to users. These human checkpoints provide patient protection and maintain technical innovation. Regarding AI use in healthcare, it is the responsibility of healthcare professionals to inform patients how their data may be used and how they can oppose data sharing, and to ensure data anonymization [112]. It is also important to ensure that healthcare professionals remain at forefront of patient management, providing a critical review of AI-based analysis, confirming results, and ultimately informing and advising patients of their diagnosis and its implications. Pilot initiatives for “Human Oversight Colleges”, bringing together designers of AI systems, professional representatives and patient representatives, have already been initiated in several therapeutic areas such as oral care, radiology, biology and oncology.

Of note, the US President has issued a landmark Executive Order to establish similar levels of regulation in the US to those described in the EU AI Act, including the principle of “human oversight and determination” [113]. Nonetheless, the legal context of this Executive Order is less firmly established than the European AI Act, which has been adopted as the new regulation of the European Parliament and of the European Council and thus will enter into force from 01 August 2024.

In parallel, on 18 January 2024, the World Health Organization (WHO) released its own AI ethics and governance guidance, including 40 recommendations for consideration “to ensure the appropriate use of LMMs to promote and protect the health of populations” [114]. This guidance raises awareness about the integration of generative AI in healthcare systems and about the ethical and human rights implications that should be handled by governments worldwide. It also highlights a pressing need for human oversight of AI.

In summary, these AI technologies generate specific ethical risks that the new AI regulatory frameworks in Europe and the US intend to address. The need for human oversight of AI is an essential issue for allowing open innovation while ensuring human involvement remains at the centre of patient care at a time of technological revolution.

### Impact of AI on the management of rare diseases

As mentioned throughout this review, AI-based techniques may be used at different time points during the management of patients with rare diseases. Providing that consent has been obtained from the patient or their representatives, genomic, physiologic, imaging and EHR data can be analysed by AI-based tools. These analyses can be conducted outside of expert rare disease centres. Facial or dermoscopic photographs, fundus examination, ECG, cMRI or brain MRI images can be processed for image recognition patterns and provide first hints to the right diagnosis. The specific signature of each clinical sign and symptom can be processed to allow for differential diagnosis from other rare or more common diseases using adapted AI-based algorithms. For most rare diseases, especially those requiring multiple clinical exams, a multimodal AI-based approach will likely help to promote faster and accurate diagnosis, initiation of appropriate therapy, and regular monitoring of specific physiological metrics to follow disease progression. The outcome of AI-based algorithms generally corresponds to a ranked list of scores and the singularity of each patient will be ultimately managed by the experts. The wide range of AI tools provides the potential for more Fabry disease patients to be diagnosed and treated earlier, thereby preventing organ impairment. Since algorithms may facilitate the analysis of patient genomes, images, and laboratory results, physicians may have more time during consultations, benefiting patient follow-up and increasing understanding of the natural history of the disease. Overall, in this review three major AI-based methods have been identified as bringing substantial improvements for the diagnosis of Fabry disease: EHR screening based on NLP, facial recognition, and cMRI-based differential diagnosis.

## Conclusions

Patient diagnostic journeys may benefit from AI as these technologies may reduce the rate of misdiagnosis and shorten the period spent without appropriate medical care, thus lessening the psychological and physiological impact of disease on health status. Raising awareness and using appropriate resources are key steps towards earlier and better diagnosis of rare diseases. The large amounts of data available from EHRs, medical imaging (standardized images) and DNA sequencing allow for large-population and patient-centred approaches, and the leveraging EHRs and the use of AI-based tools in a multimodal approach should accelerate accurate rare disease diagnosis, supporting but not replacing healthcare professionals in their expert adjudication of patient diagnosis. The rapid redirection of patients to rare disease experts via chatbots, and consultations via telemedicine should ensure faster management. The AI-based automation of certain tasks may also allow healthcare professionals to dedicate more time to patient care, most notably to tasks that require human intervention such as delivery of the diagnosis and prognosis, and support with disease management. Overall, ML- and DL-based methods have been shown to be capable of accurate data interpretation, which may be of particular benefit for non-specialists, and the implementation of these technologies in general healthcare and medical imaging centres may help to reduce the diagnostic odyssey. However, it is important to note that we are still at the early stages of the AI-based technological revolution that is transforming healthcare for both patients and professionals. It is of utmost importance that we approach this transformation with an open mind towards innovation, while maintaining the pace of positive regulation concerning the ethical stakes.

## Data Availability

As this article is a literature review, no original data were used.

## Abbreviations

AI: Artificial intelligence
ANN: Artificial neural networks
cMRI: Cardiovascular magnetic resonance imaging
CNLP: Clinical natural language processing
CNN: Convolutional neural networks
DL: Deep learning
DNA: Desoxyribonucleic acid
EHR: Electronic health record
ERT: Enzyme replacement therapy
FASTEX: Fabry stabilization index
FLAIR: Fluid-attenuated inversion-recovery
HPO: Human Phenotype Ontology
GLA: Galactosidase alpha
LLM: Large language models
ML: Machine learning
NGS: Next-generation sequencing
NLP: Natural language processing
OMIM: Online Mendelian Inheritance in Man
PRO: Patient-reported outcome
S4C: Screen4Care
SNV: Single nucleotide variant
WES: Whole exon sequencing
WGS: Whole-genome sequencing
WHO: World Health Organization
